# Dynamic and tissue-specific proteolytic processing of chemerin in obese mice

**DOI:** 10.1371/journal.pone.0202780

**Published:** 2018-08-30

**Authors:** Lei Zhao, Yasuto Yamaguchi, Wen-Jun Shen, John Morser, Lawrence L. K. Leung

**Affiliations:** 1 Stanford University School of Medicine, Department of Medicine, Division of Hematology, Stanford, CA, United States of America; 2 Veterans Affairs Palo Alto Health Care System, Palo Alto, CA, United States of America; 3 Division of Endocrinology, Department of Medicine, Stanford University School of Medicine, Stanford, CA, United States of America; State University of Rio de Janeiro, BRAZIL

## Abstract

Chemerin is a chemoattractant involved in immunity as well as an adipokine, whose activity is regulated by successive proteolytic cleavages at its C-terminus. Chemerin’s C-terminal sequence and its proteolytic cleavage sites are highly conserved between human and mouse, as well as in other species. We produced, purified and characterized different mouse chemerin forms. Ca^2+^ mobilization assay showed that the EC_50_ values for mchem161T and mchem157R were 135.8 ± 158 nM and 71.2 ± 55.4 nM, respectively, whereas mchem156S and mchem155F had a 20-fold higher potency with an EC_50_ of 4.6 ± 1.8 nM and 3.6 ± 3.0 nM, respectively, likely representing the two physiologically active forms of chemerin. No agonist activity was found for mchem154A. Similar results were obtained in a chemotaxis assay. To identify and quantify the *in vivo* mouse chemerin forms in biological samples, we developed specific ELISAs for mchem162K, mchem157R, mchem156S, mchem155F and mchem154A, using antibodies raised against peptides from the C-terminus of the different mouse chemerin forms. The prochemerin form, mchem162K, was the major chemerin form in plasma with its increase matching the increase of total plasma chemerin in obese mice. During the onset of obesity in high-fat diet fed mice, mchem156S was elevated in plasma. In contrast, mchem155F was the dominant form in epididymal fat extracts. Our study provides the first direct evidence that mouse chemerin undergoes extensive, dynamic and tissue-specific proteolytic processing *in vivo*, similar to human chemerin, underlining the importance of measuring individual chemerin forms in studies of chemerin biology in mouse models.

## Introduction

Chemerin was identified in human inflammatory fluids as a natural ligand for the orphan G protein-coupled chemokine-like receptor 1 (CMKLR1), also known as chemR23 [1,2] and functions as a chemoattractant for leukocytes expressing CMKLR1, such as plasmacytoid dendritic cells and natural killer cells. Two additional receptors bind chemerin with high affinity, chemokine receptor-like 2 (CCRL2) [3,4] and G protein–coupled receptor 1 (GPR1) [5]. CCRl2 is not a signaling receptor and only binds chemerin presenting it to its other receptors [6].

In addition to its immune functions, chemerin is an adipokine that regulates adipocyte development and metabolic functions such as glucose metabolism [7–11] in which GPR1 has been implicated [12]. Elevated levels of chemerin have been found in patients with diabetes [13–16] and fatty liver disease [15–17]. Although serum chemerin levels are elevated in obese humans and rodents, and chemerin may serve as a chemoattractant for various types of immune cells that contribute to adipose tissue inflammation commonly found with obesity, the relationship between chemerin, obesity, and energy homeostasis remains incompletely defined [18].

Chemerin, also known as retinoic acid receptor responder 2 (RARRES2), is secreted as a precursor (prochemerin) with low biological activity that terminates in humans at amino acid serine 163 (hchem163S). Prochemerin is converted into a full agonist by truncation of the last 6 amino acids at its C-terminus by proteases belonging to the coagulation, fibrinolytic, and inflammatory cascades [19–22]. The most active form of chemerin, hchem157S, can be generated either by direct cleavage of prochemerin by neutrophil-derived serine proteases (elastase or cathepsin G) or tissue-kallikrein [23], or alternatively by sequential cleavages with clotting factor FXIa or plasmin to form hchem158K followed by removal of the C-terminal lysine by carboxypeptidase N (CPN) or carboxypeptidase B2 (CPB2, also termed thrombin-activatable fibrinolysis inhibitor) producing hchem157S [20]. Enzymatic proteolysis also inactivates bioactive chemerin. Neutrophil-derived protease 3, mast cell chymase [24], and angiotensin-converting enzyme [25] can all convert active chemerin into inactive derivatives. Thus precise proteolytic processing is a key regulatory mechanism for generating local chemerin bioactivity in a tissue-specific manner.

Mice deficient in the chemerin gene or its receptor genes have been created, but there are no systematic studies of the properties of the different mouse chemerin forms. The mouse homologue (mchem156S) of human chem157S exerts anti-inflammatory activities on macrophage activation in zymosan-induced peritonitis [26]. Anti-inflammatory properties of chemerin were also observed in a LPS-induced acute lung injury model [27] and acute viral pneumonia [28]. However, *ex vivo* experiments using peritoneal macrophages from wild type ChemR23^-/-^ mice treated with thioglycollate or polyacrylamide beads failed to show inhibitory effects of chemerin on pro-inflammatory cytokine production [29].

To study chemerin biology in the mouse model, we produced and purified the different mouse chemerin forms and characterized their activities *in vitro*. We also developed specific ELISAs for each of the mouse chemerin forms. Here, we report on the activity of different mouse chemerin forms and communicate the first observations on the presence of the different chemerin forms in obese mice.

## Materials and methods

### Homology of chemerins from different species

Chemerin sequences (RARRES2) were extracted from the Ensembl database (http://www.ensembl.org/index.html) on 24^th^ October 2017 and compared using the multiple sequence alignment programs Clustal omega and Wasabi.

### Production and purification of mouse chemerins

The production and purification of mouse chemerins was carried out using similar methods to those described previously for human chemerins [30]. Mammalian Gene Collection mouse retinoic acid receptor responder 2 (RARRES2) cDNA [BC038914] was obtained from Open Biosystems (Huntsville, AL). cDNA fragments encoding various mouse chemerin forms (mchem162K, mchem157R, mchem156S, mchem155F and mchem154A) were amplified by PCR primers listed in S1 Table before cloning them into a ubiquitous chromatin opening element vector, pCET-1019AS-puro, provided by Millipore. The chemerin constructs were transfected into CHO-S cells and subsequent selection was performed [30]. For mchem162K and mchem154A cells were grown in suspension culture for 4 days at 37°C and purified [30]. For mchem156S and mchem155F, the CHO-S cell clones were cultivated in 225 cm² T-flasks for 3 days to avoid cleavage of the secreted chemerins. As mchem157R was susceptible to proteolytic cleavage in the CHO-S conditioned medium, it was generated from mchem162K by digestion with 10 milliunits of mouse plasmin (55:1 substrate:enzyme molar ratio; Haematologic Technologies Inc., Essex Junction, VT) for 10 min at 37°C. Purified proteins were characterized by SDS-PAGE, Edman N-terminal sequencing and matrix-assisted laser desorption/ionization-time of flight (MALDI-TOF) mass spectrometry (PAN Facility, Stanford University School of Medicine). Endotoxin levels in the purified proteins were determined using ToxinSensor^TM^ Chromogenic LAL Endotoxin Assay Kit (GenScript Inc., Piscataway, NJ) according to the manufacturer’s protocol.

### Chemotaxis and calcium mobilization assays

Mouse pre-B lymphoma L1.2 cells, stably expressing mouse CMKLR1 were provided by Dr. Brian Zabel and Dr. Eugene C. Butcher (Stanford University School of Medicine and Veterans Affairs Palo Alto Health Care System) and cultured in RPMI 1640 with 10% FBS and 1 mg/ml geneticin as first described [31]. Cells were treated with 5 mM sodium butyrate for 16 hrs and then used for chemotaxis and calcium mobilization assays [30]. Each assay was repeated at least three independent times for each protein.

### Antibody preparation

Preparation of antibodies specific for different forms of mouse chemerin was carried out using similar methods to those described previously for human chemerins [32]. Rabbit polyclonal antibodies anti-mchem162K and anti-mchem157R were raised against peptides derived from the C termini of the mouse chemerin sequence, ^150^CGQFAFSRALRTK^162^ (anti-mchem162K) and ^150^CGQFAFSR^157^(anti-mchem157R), that were conjugated to keyhole limpet hemocyanin (KLH) (Covance, Denver, PA) and specific antibodies purified from the antisera as described [32]. Rabbit polyclonal anti-human chemerin 157S (anti-hchem157S) [32] was used as the specific antibody against mouse chemerin 156S (anti-mchem156S). Chicken polyclonal anti-human chemerin 156F (anti-hchem156F) IgY was used as the specific antibody against mouse chemerin 155F (anti-mchem155F). Anti-human chemerin 156F chicken IgY was raised against the peptide sequence CZ^151^PGQFAF^156^ conjugated to KLH (Aves labs, Tigard, OR) and anti-chem156F IgY from eggs was purified by affinity chromatography on a column with its cognate peptide bound to sepharose. To eliminate cross-reactivity with the hchem157S form, negative selection affinity chromatography by adsorption on amino-linked (AminoLink kit, Thermo Fisher Scientific, Rockford, IL) column coupled with noncognate chemerin peptide KC^152^GQFAFS^157^ was performed. Chicken polyclonal anti-hchem155A IgY was used as the specific antibody against mchem154A [33]. Antibodies are listed in S2 Table.

### Western blot analysis of the specificity of anti-mouse chemerin IgGs and IgYs

The specificity of the anti-mouse antibodies specific for different forms of mouse chemerin was tested using similar methods to those described previously for human chemerins [32]. Purified mchem162K (mchem161T), mchem157R, mchem156S, mchem155F and mchem154A (~280 ng each) were separated by SDS-PAGE under reducing conditions followed by western blotting with purified anti-mchem162K, anti-mchem157R, anti-mchem156S IgG or anti-mchem155F and anti-mchem154A IgY (500 ng/ml each). The blots were developed with peroxidase-conjugated goat anti-rabbit IgG antibody (100 ng/ml, Jackson Immuno Research Labs, West Grove, PA) or peroxidase-conjugated goat anti-chicken IgY antibody (100 ng/ml, Aves labs, Inc. Tigard, OR) and detected using ECL (GE Healthcare).

### Specific ELISAs for mouse chemerin forms

Development of ELISAs specific for different forms of mouse chemerin was carried out using similar methods to those described previously for human chemerins [32]. A rat monoclonal anti-mouse chemerin antibody (4 μg/ml) (R&D Systems, Minneapolis, MN) in PBS buffer was coated onto 96-well ELISA plates, and nonspecific binding sites were blocked with 1% BSA in PBS for 1 hr. Purified proteins were used as standards to construct calibration curves. Samples and standards were diluted with 1% BSA in PBS and incubated for 2 hrs. After washing with 0.05% Tween 20 in PBS, samples were incubated with specific cognate antibodies (500 ng/ml) in PBS with 1% BSA for 1 hr. In the case of anti-mchem162K, 10 μg/ml mchem157R peptide was added to remove the residual cross-reactivity observed with mchem157R. In the case of anti-mchem155F, mchem154A and mchem156S peptides (10 μg/ml each) were added to remove the residual cross-reactivity with mchem154A and mchem156S. For anti-mchem154A, mchem161T and mchem155F peptides (10 μg/ml each) were added to remove the residual cross-reactivity with mchem155F and mchem161T. After washing with 0.05% Tween 20 in PBS, the samples were incubated with peroxidase-conjugated goat anti-rabbit IgG antibody (100 ng/ml) or peroxidase-conjugated goat anti-chicken IgY antibody (100 ng/ml) in PBS with 1% BSA for 1 hr. After washing, tetramethylbenzidine substrate (Alpha Diagnostic International, San Antonio, TX) was incubated for 10 min followed by the addition of Stop Solution (Alpha Diagnostic International) and measurement of absorbance at 450 nm. The concentrations of mouse chemerin forms were calculated from the calibration curves of the purified mouse chemerin standards. Total mouse chemerin was determined using with Mouse Chemerin Quantikine ELISA (R&D Systems).

### Body weight and composition of mice fed different diets by dual-energy X-ray absorptiometry (DEXA)

12-week and 26-week old male C57BL/6J DIO mice fed with a high-fat diet (60% fat diet, D12492, Research Diets, Inc., New Brunswick, NJ) or low-fat diet (10% fat diet, D12450B) were purchased from The Jackson Laboratory (Sacramento, CA). Animals were anesthetized with a ketamine (120mg/kg)/xylazine (5mg/kg) cocktail injection and body composition measured by DEXA using a Discovery model DEXA scanner adapted for rodent imaging (Hologic, Bedford, MA). All mouse experiments were carried out in accordance with NIH guidelines under protocol #1454 approved by the Palo Alto Veterans Affairs Institutional Animal Care and Use Committee.

### Detection of chemerin in plasma and adipose tissue extracts of mice fed different diets

12-week and 26-week old male C57BL/6J DIO mice fed with a high-fat diet (60% fat diet) or low-fat diet mice were anesthetized and blood was collected from the retro-orbital vein into Na-Citrate tubes, and plasma immediately prepared. Following sacrifice, adipose tissue (epididymal fat pads and brown fat pads) were isolated, and extracts (200 mg) were homogenized in 300 μl of 50 mM Tris-HCl, 8% sucrose, 1 mM EDTA, 0.1 mM Na_3_VO_4_, 50 mM NaF, pH 7.4 containing 10 μg/ml leupeptin with a homogenizer (T10 basic ULTRA-TURRAX Disperser with S10N-5G Dispersing Tool, IKA Works, Inc., Wilmington, NC) before centrifuging at 14,000 g for 15 min and collecting the infernatants using a syringe with an 18-gauge needle. The centrifugation step was repeated 1–2 times to remove all fat. Plasma and adipose tissue extracts were stored frozen at -80°C.

After thawing, plasma samples were diluted 250-fold and adipose tissue epididymal fat and brown fat extracts were diluted 10-fold in calibrator diluent and assayed by the ELISA for total chemerin. For the specific ELISAs described above, plasma samples were diluted in 1% BSA-PBS as follows: 100-fold for mouse chem162K/161T; 15-fold for mouse chem157R; and 1–3 fold for mouse chem156S, 155F and 154A. Mouse epididymal fat extracts were assayed without dilution.

### Detection of adiponectin in mouse plasma and adipose tissue extracts

After thawing, plasma was diluted 2000-fold while both epididymal fat and brown fat extracts were diluted 1000-fold in assay buffer and assayed in the Mouse Adiponectin/Acrp30 Quantikine ELISA (R&D Systems) for adiponectin.

### Determination of protein and DNA concentrations in adipose tissue extracts from mice

Epididymal fat extracts were diluted 50-fold and brown fat extracts diluted 100-fold in assay buffer and assayed by BCA assay (Bio-Rad, Hercules, CA) to determine total protein concentration. Both epididymal fat and brown fat extracts were diluted 50-fold in assay buffer and assayed by Quanti-iT PicoGreen dsDNA assay kit (Invitrogen, Carlsbad, CA) for total DNA concentration. Data were normalized to μg DNA.

### Statistical analysis

Values for 50% effective concentration (EC_50_) were determined using nonlinear regression applied to a sigmoidal dose-response model. Comparison of two groups was by Student’s t test; multigroup comparisons were analyzed by ANOVA followed by Kruskal-Wallis analysis. Correlation analysis was calculated by Pearson analysis with two-tailed p value. The statistical analysis employed Prism v7 (GraphPad, La Jolla, CA). Values of *p* < 0.05 were considered significant.

## Results

### Chemerin homology

**Fig 1:**
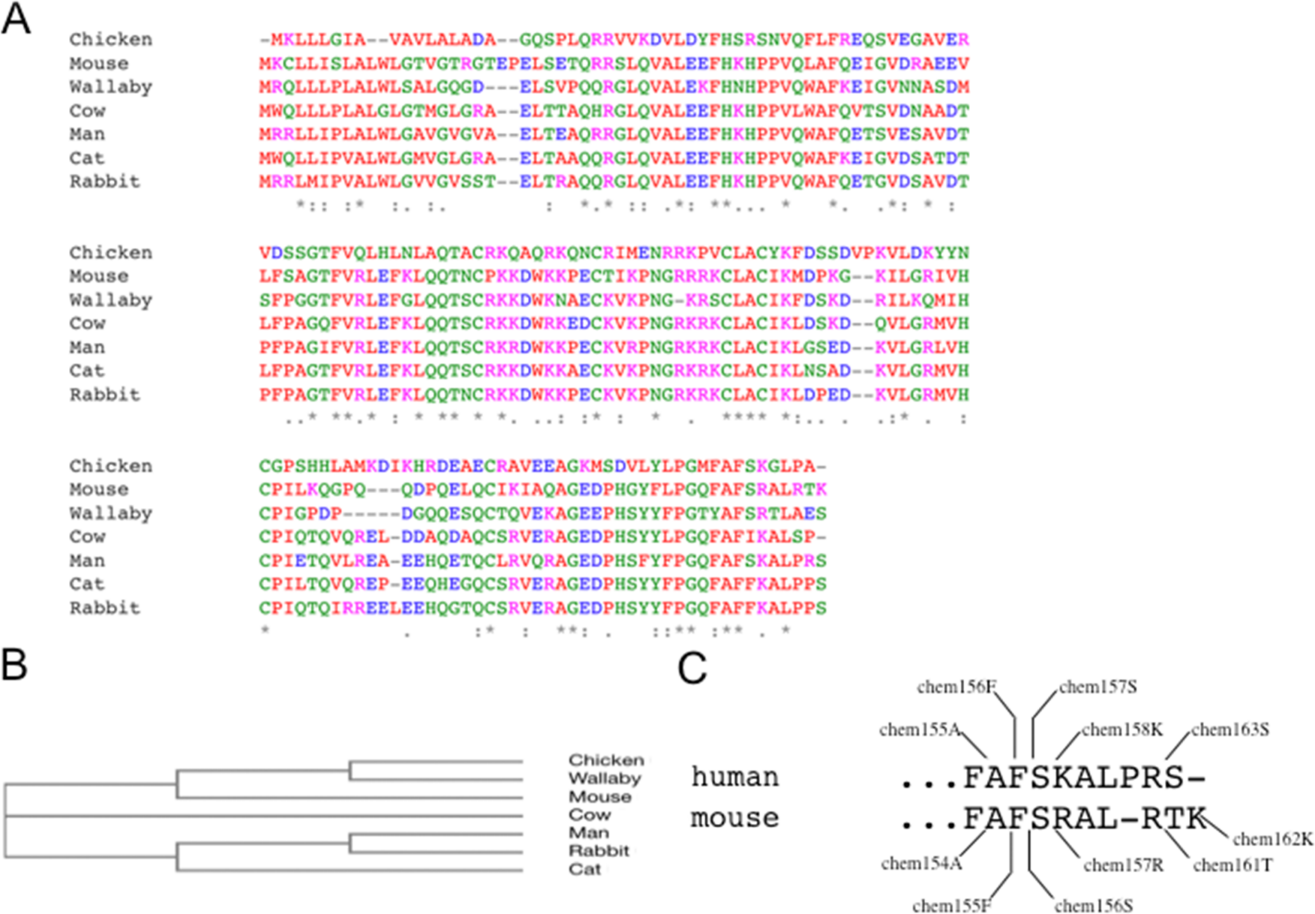
Chemerin homology. **(A)** alignment of chemerin protein sequences from 9 species. **(B)** dendrogram showing an evolutionary tree of those species. **(C)** human and mouse chemerin C-terminal peptides. Chemerin sequences (RARRES2) were extracted from the Ensembl database and compared using Clustal omega.

To explore the importance of the C-terminal residues that had previously been identified as key for activation and inactivation of human chemerin, we investigated the sequence homology of chemerins from all species in which the chemerin gene could be identified in the ENSEMBL database. Alignment of chemerin protein sequences from species representing several mammalian orders as well as a bird (chicken, Gallus gallus) which has two copies of the chemerin gene and an amphibian (Xenopus tropicalis) showed good conservation between the different species (Figs 1A and S1). There is homology throughout the protein including the conservation of the six Cys residues. The C-terminal sequence responsible for activity in human chemerin, ^148^(Y,F)(F,L)PG(**M**,Q)FAF(S,I,F)(K,R)(A,**G,**T)^159^ (human numbering with alternatives at the same position shown in brackets), is very highly conserved with residues in bold only occurring in chicken chemerin. A dendrogram showing the similarity in relationships of the sequences surprisingly does not recapitulate an evolutionary tree (Fig 1B). This distribution implies that the chemerin gene appeared in the tetrapod lineage before the last common ancestor of amphibians, birds and mammals diverged.

Overall there is 64% identity between the human and mouse chemerin sequences, with 78% similarity. Human prochemerin (hchem163S) has no or low chemerin activity, hchem158K has modest activity, and hchem157S is most active; the peptide representing the C-terminus of chem156F is less active than human hchem157S, while human hchem155A is inactive [30]. In all species there is a conserved basic amino acid (in human K158) C-terminal to the form that is predicted to be active (human S157) strongly suggesting that a member(s) of the serine protease family with specificity for basic amino acids is responsible for the cleavage of the precursor. Homologous sites are present in the mouse chemerin sequence (Fig 1C) and we chose to produce those five chemerin forms.

### Production of mouse chemerin forms

Mouse chemerin forms were produced by the methods we had previously developed for human chemerins [30]. The productivity of the clones expressing different mouse chemerin forms (Table 1) was 9.19, 1.20, 8.49, 6.63, and 7.81 pg/cell per day for mchem162K, mchem157R, mchem156S, mchem155F and mchem154A respectively.

**Table 1:** Properties of chemerin forms.

	C-terminal Sequence	Amino Acids	Observed Mass	Molecular Mass	pI	Ca^2+^ flux (nM)	Chemotaxis (nM)
mchem161T	GYFLPGQFAFSRALRT	142	16208.9 ± 1.8	16344.9 (mchem162K)	9.11	135.8 ± 158.0	156.9 ± 116.8
mchem157R	GYFLPGQFAFSR	138	15765.2 ± 2.3	15765.2	8.96	71.2 ± 55.4	156.2 ± 11.4
mchem156S	GYFLPGQFAFS	137	15609.8 ± 2.5	15309.1	8.77	4.6 ± 1.8	11.8 ± 2.8
mchem155F	GYFLPGQFAF	136	15519.0 ± 2.9	15522	8.77	3.6 ± 3.0	9.8 ± 5.9
mchem154A	GYFLPGQFA	135	15374.5 ± 1.9	15374.8	8.77	>1000	>1000

The C-terminal sequences of all chemerin forms tested in this study are displayed, as well as the EC_50_ values on mouse CMKLR1/L1.2 cells determined by Ca^2+^ mobilization and chemotaxis assays. Data are shown as mean ± S.D. and calculated from at least three independent experiments.

Mouse chemerins were purified by single-step cation exchange chromatography and purity determined by SDS-PAGE (S2 Fig). Interestingly, mouse chemerin proteins eluted earlier than the equivalent human chemerins despite their calculated pIs being slightly higher than the corresponding human chemerin forms. Edman sequencing of the purified proteins confirmed that all had the same uniform N terminus, TEPELSETQR. Analysis of the proteins by MALDI-TOF-MS showed that the observed masses for mchem157R, mchem156S, mchem155F and mchem154A were almost identical to those estimated from their sequence (Table 1 and S3 Fig). However, the observed mass for mchem162K was 16208.6, which was 126.3 daltons smaller than the predicted molecular mass of 16334.9, suggesting that mchem162K was missing the C-terminal lysine and terminated at T^161^. We were unable to identify conditions that allowed production of mchem162K. Therefore, we used mchem161T as mouse prochemerin in our studies. Under all culture conditions tested, the conditioned medium for mchem157R contained not only mchem157R but also significant amounts of mchem154A. Consequently we produced mchem157R by plasmin cleavage of mchem161T [30].

### Functional characterization of mouse chemerin forms

**Fig 2:**
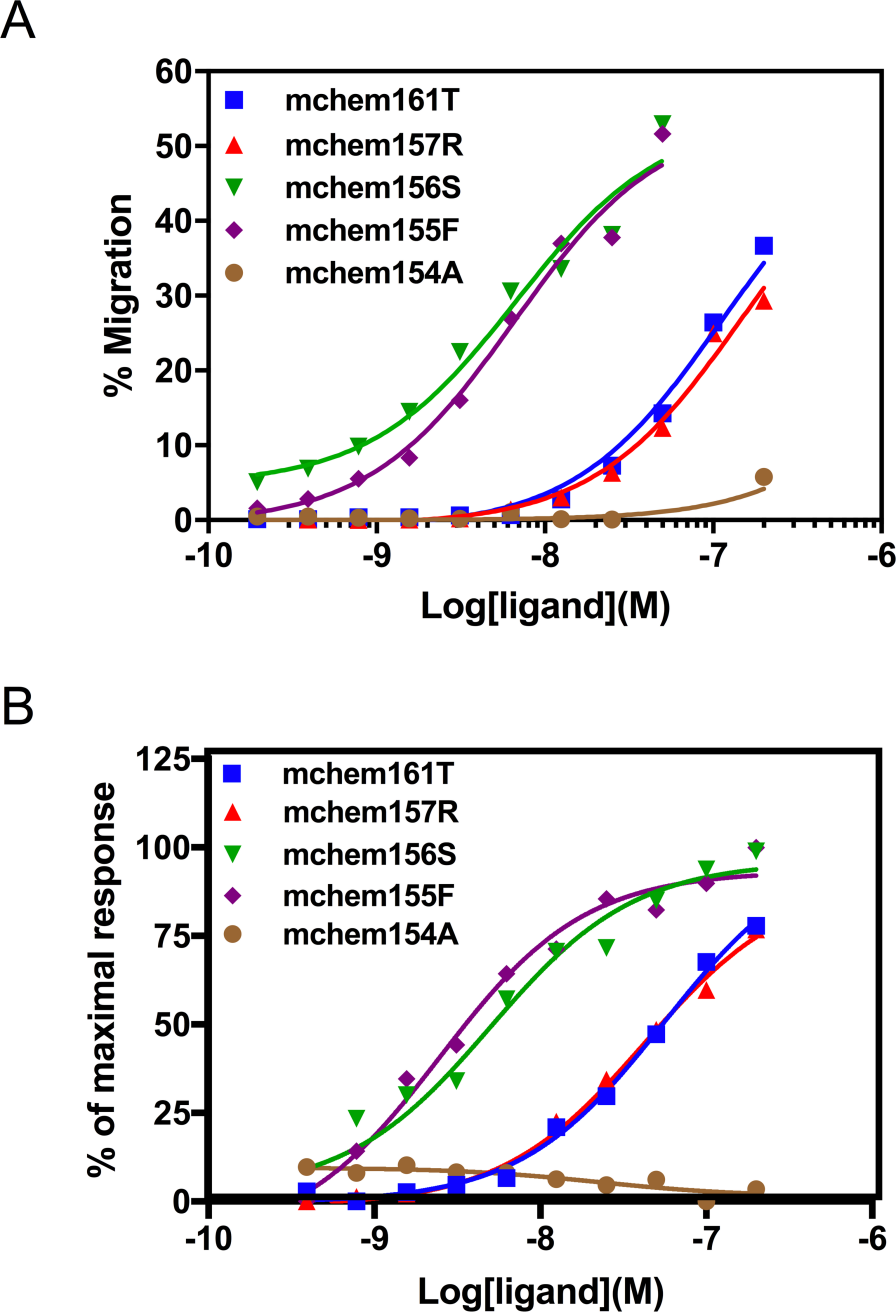
Biological activities of mouse chemerin forms. **(A)** the indicated concentrations of mchem161T(*blue*), mchem157R(*red*), mchem156S(*green*), mchem155F(*purple*), and mchem154A (*brown*) were assayed for their chemotactic activity on mCMKLR1/L1.2 cells. **(B)** Calcium flux in mCMKLR1/L1.2 cells in response to these proteins.

We evaluated the functional properties of the purified chemerins using L1.2 cells expressing mouse CMKLR1. Chemotaxis assays showed that the EC_50_ values for mchem161T and mchem157R were 156.9 ± 116.8nM and 156.2 ± 11.4 nM, respectively, while mchem156S had about 15-fold higher potency with an EC_50_ of 11.8 ± 2.8 nM. mchem155F was as potent as mchem156S, with an EC_50_ of 9.8 ± 5.9 nM. On the other hand, mchem154A had low activity (Table 1 and Fig 2A).

To eliminate the possibility of overestimating the potency due to additional processing of the proteins into more potent forms during the chemotaxis assay, the proteins were tested for their ability to trigger a transient release of intracellular calcium (Table 1 and Fig 2B). EC_50_ values for mchem161T and mchem157R were 135.8 ± 158.0 nM and 71.2 ± 55.4 nM, respectively. In contrast, both mchem156S and mchem155F were 20-fold more potent (EC_50_ of 4.6 ± 1.8 nM and 3.6 ± 3.0 nM respectively). In the calcium mobilization assay, mchem154A had no detectable activity when tested at concentrations up to 2 μM. Thus, similar results were obtained in both assays, demonstrating that mchem161T behaved as a precursor and proteolytic processing to generate mchem156S or mchem155F is necessary for maximal activity.

### Characterization of specific antibodies directed against different mouse chemerin forms

**Fig 3:**
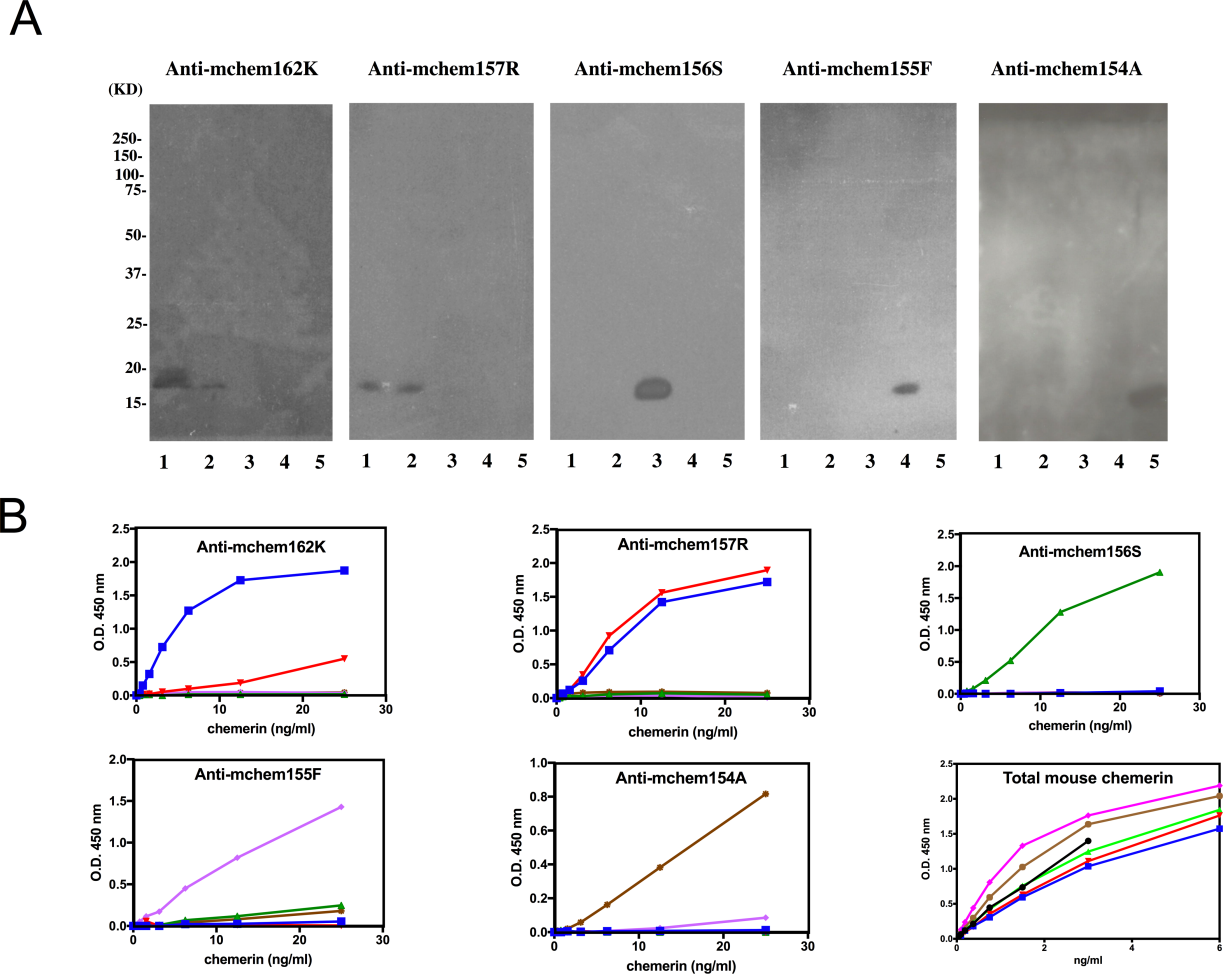
Characterization of specific antibodies against chemerin forms. **(A)** purified antibodies specific for different chemerin forms were characterized by western blot analysis of mchem161T (lane 1), mchem157R (lane 2), mchem156S (lane 3), mchem155F (lane 4), and mchem154A (lane 5) with anti-mchem162K, anti-mchem157R, anti-mchem156S, anti-mchem156F, and anti-mchem154A. Molecular mass markers are shown on the left of the panels. **(B)** mchem161T (*blue*), mchem157R (*red*), mchem156S (*green*), mchem155F (*purple*) and mchem154A (*brown*) were detected with specific ELISAs using anti-mchem162K, anti-mchem157R, anti-mchem156S, anti-mchem155F, and anti-mchem154A respectively. Bottom right panel displays the results of mchem161T (*blue*), mchem157R (*red*), mchem156S (*green*), mchem155F (*purple*) mchem154A (*brown*) and R&D standard (*black*) in the R&D total mouse chemerin ELISA.

Because the activity of the different mouse chemerin forms varies significantly, measurements of the overall levels of chemerin in biological fluids would not give an accurate description of the status of the chemerin system. We therefore developed a panel of ELISAs to specifically detect individual chemerin forms in mouse samples [32]. The specificity of these anti-chemerin antibodies was demonstrated by western blotting in which each antibody only recognized its cognate protein and not the other four chemerin forms, except for anti-mchem157R, which recognized both mchem161T and mchem157R despite having undergone negative depletion (Fig 3A).

### Development of ELISAs specific for different mouse chemerin forms

We used these antibodies to develop sandwich ELISAs specific for mchem162K/161T, mchem157R, mchem156S, mchem155F and mchem154A. mchem161T, but not mchem157R, mchem156S, mchem155F, or mchem154A, was detected in a dose-dependent manner by anti-mchem162K specifically, with a lower limit of detection of 0.2 ng/ml (Fig 3B). Even though the protein that we used was mchem161T, instead of mchem162K, the anti-mchem162K antibody gave a good dose-response curve, indicating that mchem161T could be used as a standard for mchem162K. Anti-mchem156S, anti-mchem155F and anti-mchem154A specifically recognized their cognate ligands and not the other forms. On the other hand, anti-mchem157R recognized both mchem161T and mchem157R and not the other three forms (Fig 3B). Thus, similar results were obtained in both western blotting and ELISA analysis, confirming that the anti-mchem157R antibody cross-reacted strongly with mchem162K/161T.

To determine mchem157R, as the antibody reacts equivalently with mchem161T and mchem157R, we used the signal from the anti-mchem157R ELISA to give the total concentration of [mchem162K/161T + mchem157R] and then subtracted the mchem162K/161T concentration, measured by the specific mchem162K/161T ELISA, to derive the concentration of mchem157R. Using this panel of ELISAs, the levels of different forms of mouse chemerin could be determined specifically.

The reactivity of the different chemerin forms was tested in the commercial total ELISA kit. Similar to the human situation [33], the various mouse chemerin forms had EC_50_ values that varied by more than two-fold (chem162K: 6.3 ng/mL; chem157R: 5.2 ng/mL; chem156S: 4.6 ng/mL; chem155F: 2.3 ng/mL and chem154A: 3.1 ng/mL). The standard supplied with the kit is chem156S produced in E. coli with an EC_50_ of 4.5ng/mL, which is very similar to chem156S produced in mammalian culture. Thus measurements of total chemerin in mouse biopsy samples using the commercial kit will underestimate the chemerin levels in samples if they contain chem162K and chem157R and overestimate them if they contain chem155F or chem154A.

### High-fat diet fed mice weighed more and had a higher % of fat

**Fig 4:**
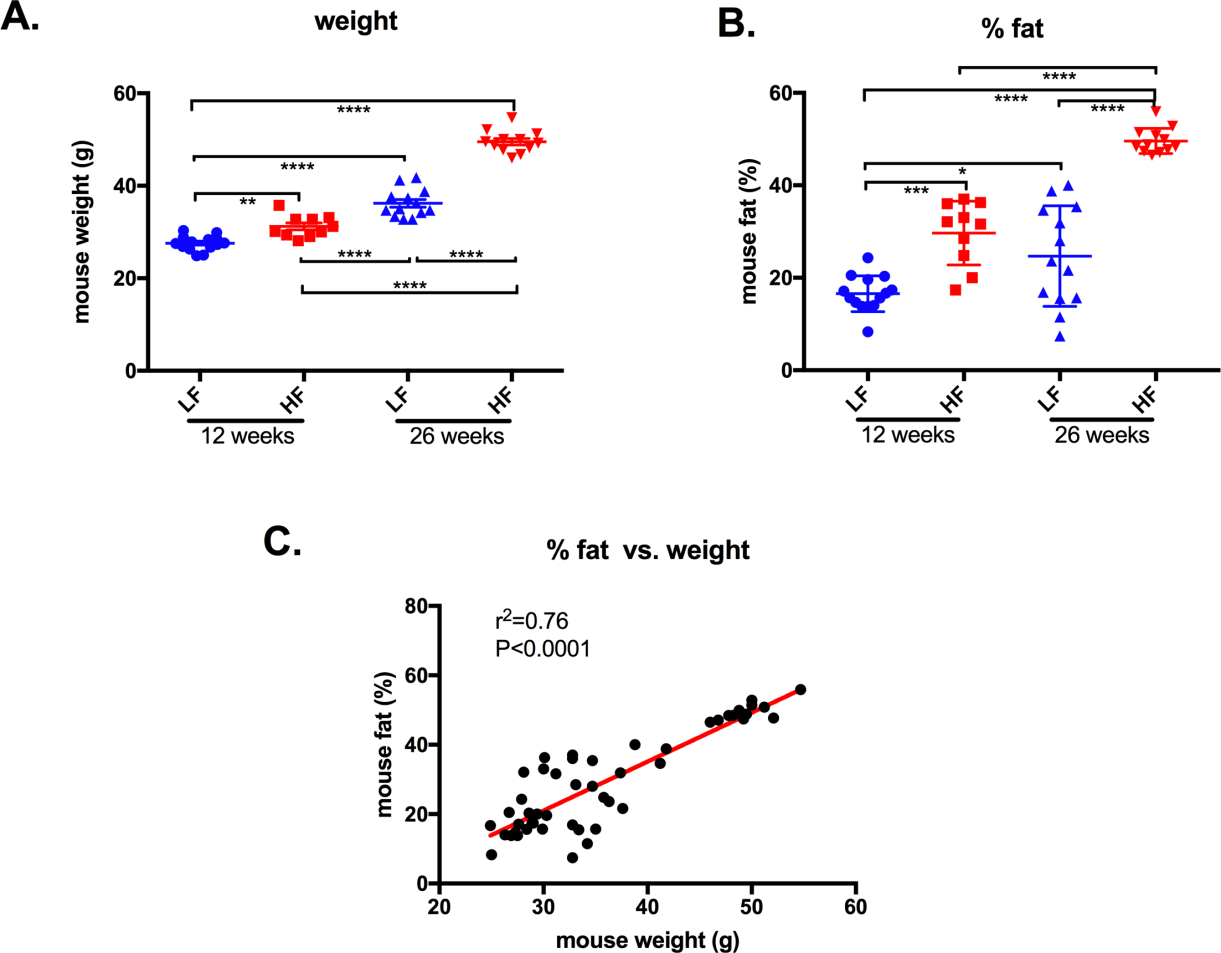
Weight and body fat % of mice fed with a low-fat or high-fat diet. Weight **(A)** and body fat % **(B)** of 12 weeks old mice fed with either a LF (designated with a blue circle) or HF (designated with a red square) diet, and 26 weeks old mice fed with either a LF (designated with a blue triangle) or HF (designated with a red triangle) diet were determined by DEXA scanner. Horizontal lines show the mean ± SEM. (n = 10–14). Multigroup comparisons were by ANOVA followed by Kruskal-Wallis analysis. *: p<0.05; **: p<0.01; *** p<0.001; ****: p<0.0001. **(C)** relationship between mouse body fat % and weight (n = 49), The Pearson correlation coefficient was calculated with a two-tailed p value.

Male mice (12- and 26-week old) were weighed and scanned by DEXA to determine their body’s fat percentage. As expected, mice fed the high-fat diet were heavier than low-fat diet mice at both time points while the percentage of fat was increased in high-fat diet mice, reaching ~50% by 26 weeks. Mouse weight and body fat percentage were positively correlated (Fig 4).

### Specific chemerin forms in low-fat and high-fat diet-fed mouse plasma

**Fig 5:**
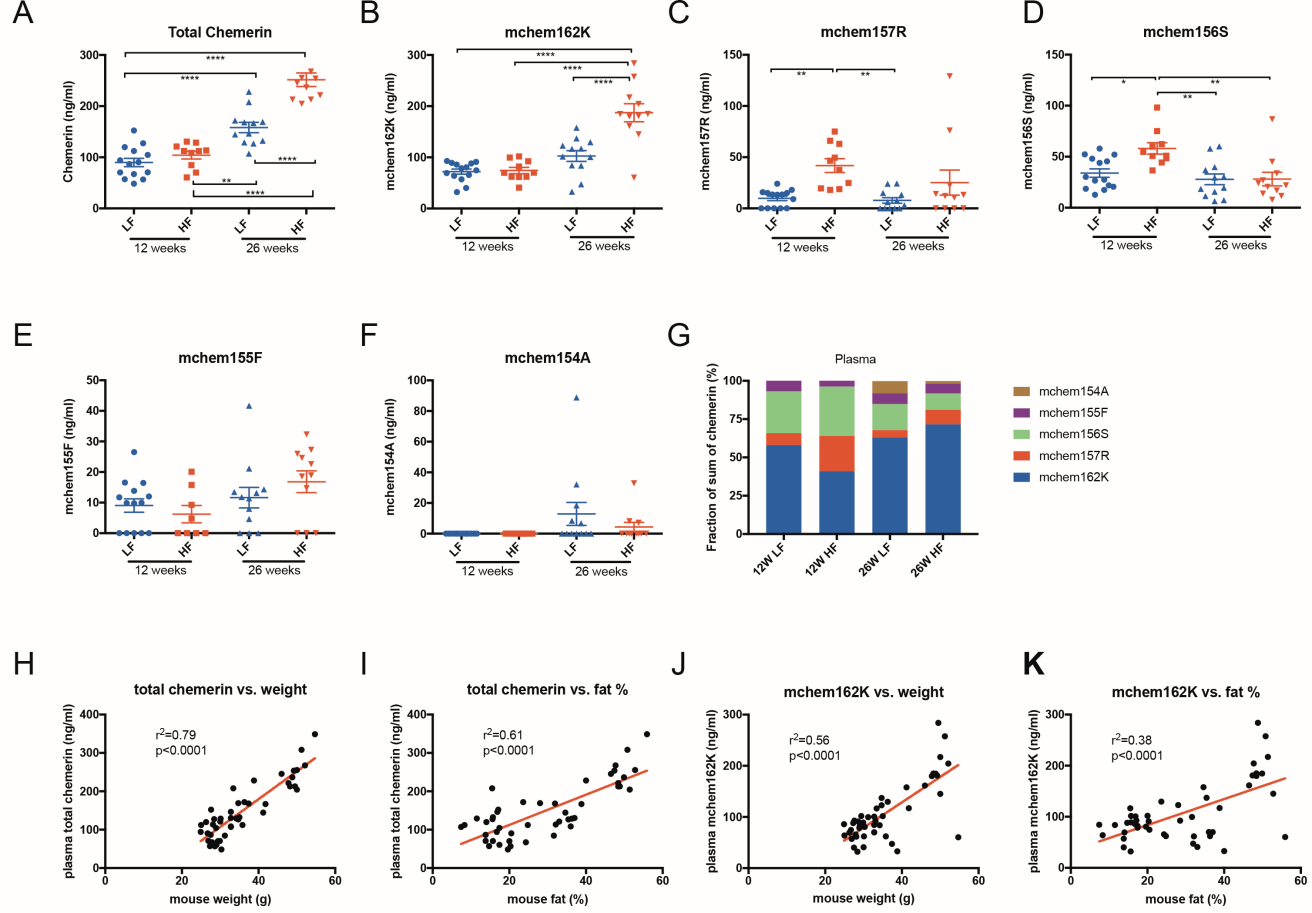
Chemerin levels in plasma of mice fed a low-fat or high-fat diet. Total chemerin **(A)**, mchem162K **(B)**, mchem157R **(C)**, mchem156S **(D)**, mchem155F **(E)**, and mchem154A **(F)** levels in plasma of 12 weeks old mice fed with either a low-fat (LF) (designated with a blue circle) or high-fat (HF) (designated with a red square) diet, and 26 weeks old mice fed with either a LF (designated with a blue triangle) or HF (designated with a red triangle) diet were determined using total chemerin ELISA and specific ELISAs. Horizontal lines show the mean ± SEM. (n = 10–14). **(G)** chemerin forms in plasma presented as a percentage of the sum of the five specific ELISAs (mchem162K + mchem157R + mchem156S + mchem155F + mchem154A). Comparisons were by ANOVA followed by post-hoc Kruskal-Wallis analysis with two-tailed p value. *: p<0.05; **: p<0.01; ****: p<0.0001. Relationship between mouse plasma total chemerin and weight **(H)**, plasma total chemerin and body fat % (**I**), plasma mchem162K and weight **(J)**, and plasma mchem162K and body fat % **(K)** (n = 47). The Pearson correlation coefficient was calculated with a two-tailed p value.

We used the specific ELISAs to determine the plasma levels of the different chemerin forms in mice that had been fed with either low-fat or high-fat diets (summarized in Table 2). Total chemerin levels in the 26-week groups were significantly higher than those of the 12-week groups with the highest levels found in 26-week high-fat diet mice (Fig 5A). Prochemerin (mchem162K/161T) was the major chemerin form in plasma with its increase in different mouse groups matching the increase in plasma total chemerin levels (Fig 5B). The partially active chemerin form, mchem157R, was significantly higher in 12-week high-fat mice where it comprised ~23% of all chemerin forms than in low-fat mice, and slightly higher than in the 26-week high fat mice (Fig 5C). Meanwhile, the active chemerin form, mchem156S, was significantly higher in 12-week high-fat mouse group (32% of all chemerin forms) than in any other mouse group, including the 26-week high-fat mice (Fig 5D). A small amount of the other active chemerin form mchem155F (≤7.7% of all chemerin forms) was found, but there was no significant difference between groups (Fig 5E). The inactive chemerin form chem154A was only detected in plasma from older mouse at a low level (<8% of all chemerin forms; Fig 5F). Thus, the combination of mchem157R and mchem156S was significantly elevated in young high-fat fed mouse plasma but not in older mouse plasma (55.2% vs. 20.3% of all chemerin forms, Fig 5G).

**Table 2:** Chemerin forms in plasma of mice fed with low-fat or high-fat diet.

	12 weeks LF (n = 14)	12 weeks HF (n = 10)	26 weeks LF (n = 12)	26 weeks HF (n = 11)
mchem162K/161T	72.1 ± 18.9 (58%)	74.3 ± 19.5 (41%)	102.7 ± 36.2 (63%) ****	187.3 ± 58.5 (71.5%) ****, ####, ¶¶¶¶
mchem157R	9.8 ± 8.2 (7.9%)	41.9 ± 21.4 (23%) **	7.8 ± 9.3 (4.8%) ##	25.2 ± 40.7 (9.6%)
mchem156S	33.9 ± 15.4 (27.3%)	58.1 ± 17.6 (32.2%) *	27.8 ± 18.2 (17%) ##	28.1 ± 22.1 (10.7%) ##
mchem155F	9.0 ± 8.2 (7.2%)	6.2 ± 8.0 (7.7%)	11.6 ± 11.6 (7.1%)	16.8 ± 11.8 (6.4%)
mchem154A	0 (0%)	0 (0%)	12.9 ± 25.9 (7.9%)	4.4 ± 9.9 (1.7%)
Sum of all forms	124	180.5	162.8	261.8
Total chemerin	89.8 ± 30.5	104.2 ± 24.2	158.2 ± 34.7 ****, ##	251.6 ± 43.9 ****, ####, ¶¶¶¶

Plasma chemerin forms and total chemerin were determined using specific ELISA and total chemerin ELISA as depicted in Fig 5. Data are shown as ng/mL mean ± SD, (%) shows specific chemerin forms presented as a percentage of the sum of the five specific forms.

*: p<0.05;

**: p<0.01;

****: p<0.0001 vs. 12 weeks LF,

##: p<0.01;

####: p<0.0001 vs. 12 weeks HF,

¶¶¶¶: p<0.0001 vs. 26 weeks LF.

Both plasma total chemerin and prochemerin (mchem162K/161T) correlated with increases in weight and body fat (Fig 5H–5K).

### Chemerin in mouse adipose tissue

**Fig 6:**
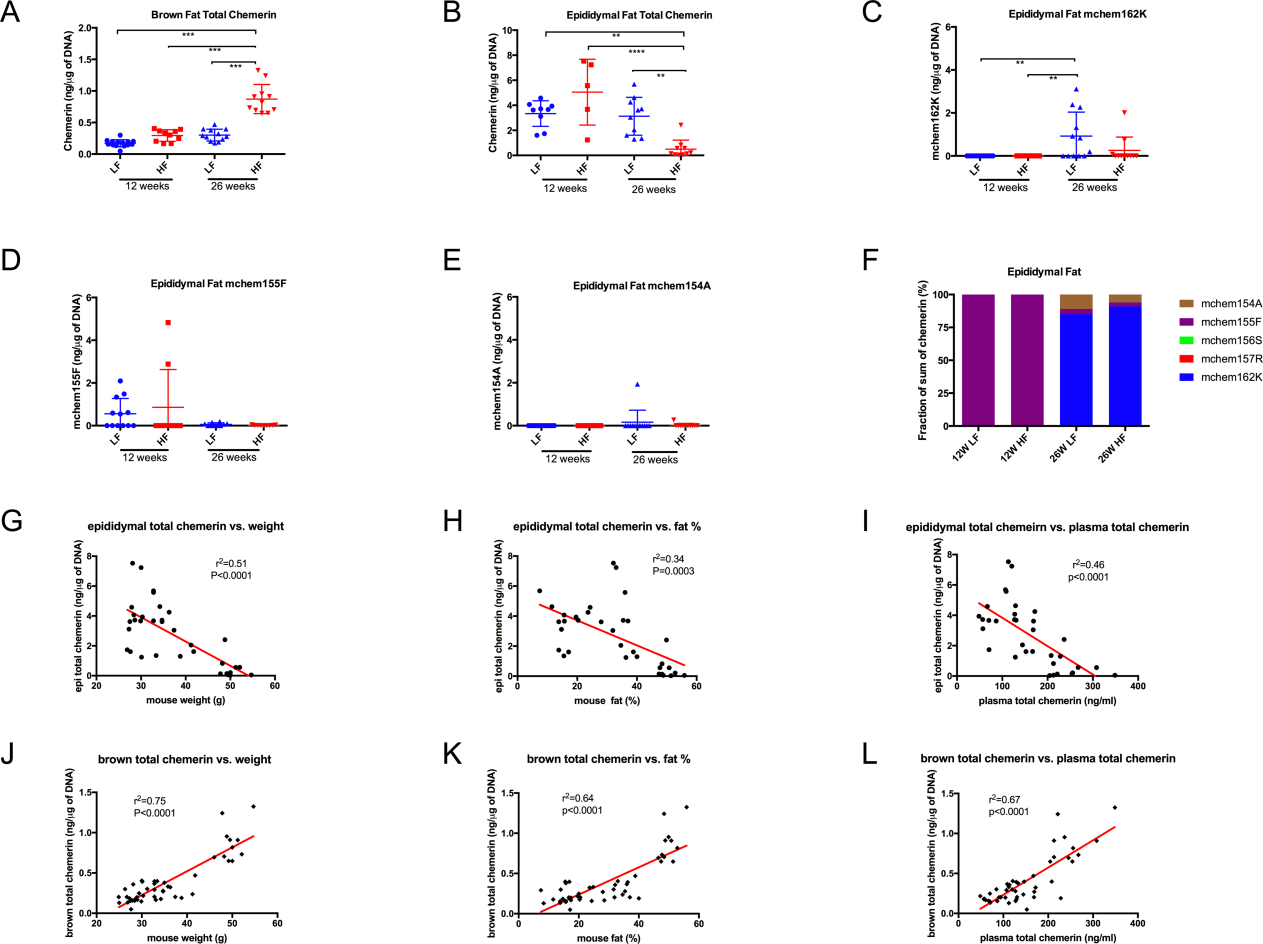
Chemerin levels in adipose tissue extracts of mice fed a low-fat or high-fat diet. Total chemerin in brown fat extracts **(A)** and epididymal fat extracts **(B)**, mchem162K, mchem155F and mchem154A in epididymal fat extracts **(C**, **D**, and **E)** of 12 weeks old mice fed with either a LF (designated with a blue circle) or HF (designated with a red square) diet, and 26 weeks old mice fed with either a LF (designated with a blue triangle) or HF (designated with a red triangle) diet were determined by ELISA. Horizontal lines show the mean ± SEM. (n = 10–14). There was no mchem157R and mchem156S detected in epididymal fat extracts. **(F)** chemerin forms in epididymal fat extracts presented as a percentage of the sum of the five specific ELISAs. Relationship between epididymal fat extracts total chemerin and weight (**G**), body fat % **(H)**, or plasma total chemerin **(I)**, brown fat extracts total chemerin and weight **(J)**, body fat % **(K)**, or plasma total chemerin **(L)** (n = 47), multigroup comparisons were by ANOVA followed by Kruskal-Wallis analysis. **: p<0.01; *** p<0.001 ****: p<0.0001. The Pearson correlation coefficient was calculated with a two-tailed p value.

Total chemerin was measured in brown and epididymal fat extracts of adipose tissue (Fig 6A and 6B and Table 3). Brown fat extracts had significantly lower total chemerin per cell than epididymal fat extracts for 12-week high-fat and low-fat fed groups, and 26-week low-fat fed group, except for 26-week high-fat fed group. While 12-week high-fat fed mice had a high level of total chemerin in epididymal fat extracts, there was a marked drop in its level by 26 weeks. On the other hand, the 26-week high-fat diet mice had significantly higher levels of chemerin in brown fat extracts than the other groups. Specific ELISAs were used to measure different chemerin forms in epididymal fat extracts. It is striking that the fully active mchem155F, but not the other active form mchem156S, dominated in 12-week mice, irrespective of the diet (Fig 6C–6F), and this dominance was lost by 26 weeks, when prochemerin comprised 85–91% of chemerin (Fig 6C–6F). No chem157R or chem156S was detected. All of the chemerin detected in 12-week mice could be accounted for by chem156F since the total chemerin ELISA overestimates chem156F. Since the total chemerin in brown fat extracts was low, we were unable to measure specific chemerin forms in them as they were below the limit of detection.

**Table 3:** Total chemerin and adiponectin in mice fed with low-fat or high-fat diet.

	12 weeks LF (n = 14)	12 weeks HF (n = 10)	26 weeks LF (n = 12)	26 weeks HF (n = 11)
Total chemerin (ng/μg of DNA) in epididymal fat extract	3.3 ± 1.0	5.1 ± 2.6	3.1 ± 1.5	0.5 ± 0.7 **, ####, ¶¶
Total chemerin (ng/μg of DNA) in brown fat extract	0.2 ± 0.1	0.3 ± 0.1	0.3 ± 0.1	0.9 ± 0.2 ***, ###, ¶¶¶
Adiponectin (ng/μg of DNA) in epididymal fat extract	613.1 ± 116.4	938.3 ± 303.3 *	523.8 ± 250.4 ##	140.9 ± 80.1 ****, ####, ¶¶¶
Adiponectin (ng/μg of DNA) in brown fat extract	172.5 ± 27.7	170.2 ± 55.8	254.6 ± 32.1 ***, ###	196.6 ± 29.0 ¶¶

Total chemerin and adiponectin in epididymal and brown fat extracts were determined by ELISA. Data are shown as mean ± SD, and normalized as ng/μg of DNA.

*: p<0.05;

**: p<0.01

***; p<0.001:

****; p<0.0001 vs. 12 weeks LF,

##: p<0.01;

####: p<0.0001 vs. 12 weeks HF,

¶¶: p<0.01;

¶¶¶: p<0.001;

¶¶¶¶: p<0.0001 vs 26 weeks LF.

Total chemerin in epididymal fat extracts is significantly decreased with increasing weight (r^2^ = 0.51, p<0.0001), fat% (r^2^ = 0.34, p<0.0001) and plasma total chemerin (r^2^ = 0.46, p<0.0001; Fig 6G–6I). In contrast, in brown fat extracts total chemerin is significantly increased with increasing weight (r^2^ = 0.75, p<0.0001), fat% (r^2^ = 0.64, p<0.0001), and plasma total chemerin (r^2^ = 0.67, p<0.0001; Fig 6J–6L).

### Adiponectin in plasma and adipose tissue extracts of mice

**Fig 7:**
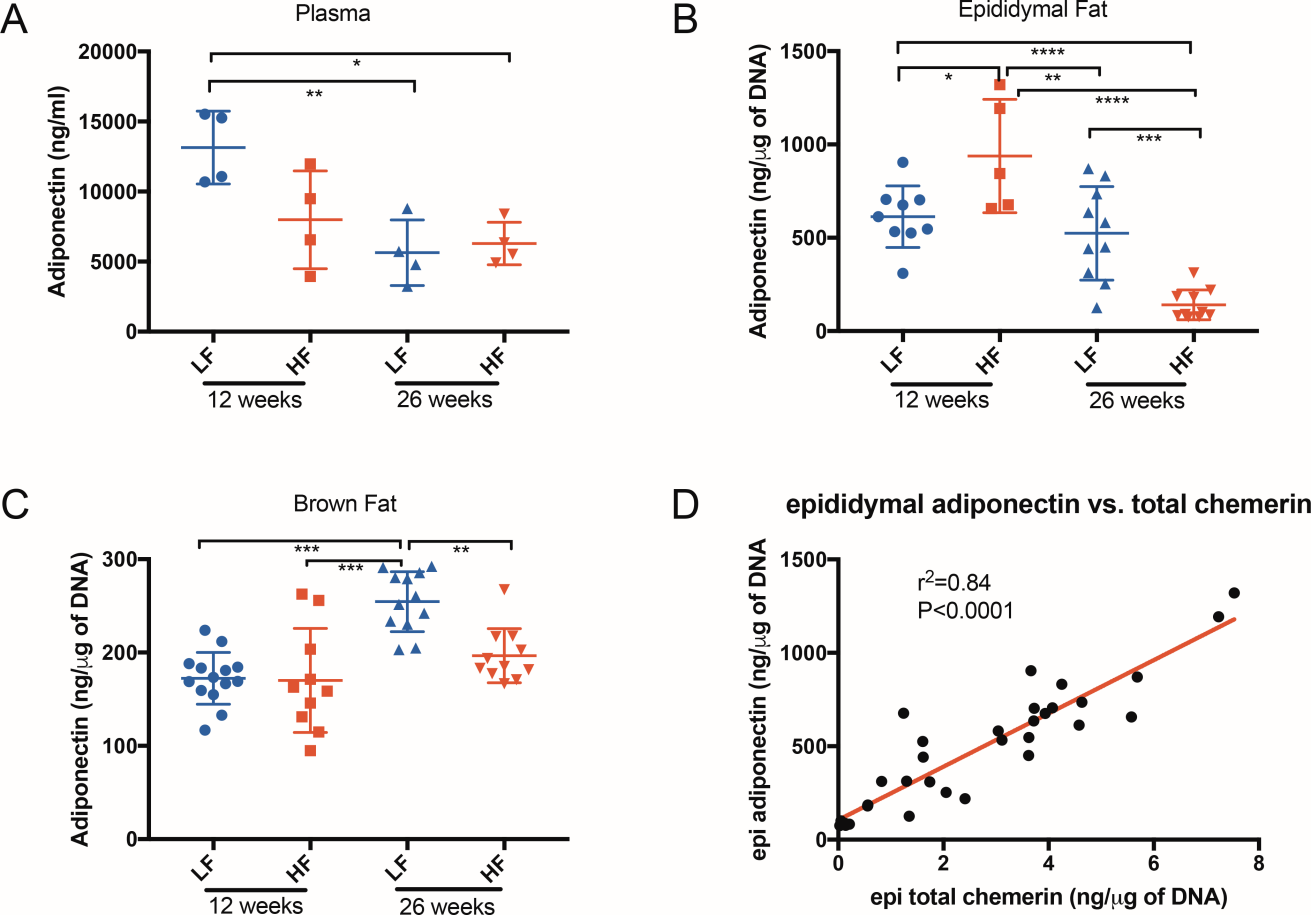
Adiponectin levels in plasma and adipose tissue extracts of mice fed a low-fat or high-fat diet. Adiponectin in plasma **(A)**, epididymal fat extracts **(B)**, and brown fat extracts **(C)** of 12 weeks old mice fed with either a LF (designated with a blue circle) or HF (designated with a red square) diet, and 26 weeks old mice fed with either a LF (designated with a blue triangle) or HF (designated with a red triangle) diet were determined with the adiponectin ELISA. Horizontal lines show the mean ± SEM. (n = 4 for **A**, n = 5–11 for **B**, and n = 10–14 for **C**). Multigroup comparison was by ANOVA followed by Kruskal-Wallis analysis. *: p<0.05; **: p<0.01; *** p<0.001 ****: p<0.0001. **(D)** relationship between adiponectin and total chemerin in epididymal fat extracts (n = 30). The Pearson correlation coefficient was calculated with a two-tailed p value.

An adipose tissue marker that varies inversely with body mass index [34], adiponectin, was measured in plasma and epididymal and brown fat extracts (Fig 7A–7C and Table 3 based on ELISA data from Figs 6 and 7). At 12 weeks, the low-fat group had a significantly higher plasma adiponectin level than the high-fat group, but it dropped with time and by 26 weeks, the plasma levels were equal in both groups. Epididymal fat extracts had higher adiponectin levels than brown fat extracts except for the 26-week high-fat diet mouse group. For epididymal fat extracts, the adiponectin level in 12-week high-fat diet mice was significantly higher than in other groups, but it dropped significantly by 26 weeks such that the level was the lowest among the four groups. In brown fat extracts, the adiponectin level in 26-week low-fat diet mice was significantly higher than in other mouse groups. Total chemerin in epididymal fat extracts was positively correlated with adiponectin levels (Fig 7D).

## Discussion

The conservation of the C-terminal sequence in chemerin, specifically with a conserved basic amino acid delineating the site of proteolytic cleavage, not just between mouse and human, but also in birds and amphibians suggests that the regulation of chemerin activity by proteolytic processing has also been conserved. In this study, we produced and purified different mouse chemerin forms using the methods we had previously developed for human chemerins [30] allowing us to fully characterize them. The most potent forms are mchem156S and mchem155F and thus probably the physiologically active forms. Prochemerin, mchem162K, which was purified as mchem161T although the plasmid encoded for chem162K, has very low activity while mchem157R is modestly active. The smaller form, mchem154A, is inactive with no detectable agonist activity.

The activity profile of the different forms of mouse chemerin is very similar to that of human chemerin forms except for the mchem155F form. hchem157S, the human homologue of mchem156S, is the most potent human chemerin form with its EC_50_ comparable to that of mchem156S [30]. The human homologue of mouse mchem155F, hchem156F, on the other hand, is much less potent than hchem157S, while mchem155F is as potent as mchem156S and hchem157S. The possible reasons for hchem156F and mchem155F to display different activities on cells expressing their cognate CMKLR1 are under investigation [35]. The activity of both mchem162K (mchem161T), the full-length mouse prochemerin form, and mchem157R was modest, analogous to their homologues, human hchem163S and hchem158K. No agonist activity was detectable either with mchem154A or hchem155A. Our data demonstrate that a similar pattern of proteolytic cleavages regulates both mouse and human chemerin activation and its subsequent inactivation, consistent with the control of chemerin activity being via proteolysis at its C-terminus.

Since the biological activity of the different mouse chemerin forms varies significantly, measurements of the overall levels of chemerin in biological fluids in previous studies do not give an accurate description of the status of the chemerin. Our panel of mouse chemerin ELISAs allows determination of the levels of the five key chemerin forms in the mouse system for the first time. Importantly, the sensitivity of each of the ELISAs is ~0.2 ng/ml (~0.012 nM) whereas the EC_50_ for the most active forms, mchem156S and mchem155F, is 4.6 ± 1.8 nM and 3.6 ± 3.0 nM, respectively. Thus, the range of the ELISAs covers the concentrations over which chemerin is active.

Using these ELISAs, we measured the plasma levels of the different chemerin forms in mice fed low-fat or high-fat diets. The prochemerin form, mchem 162K/161T, with low chemerin activity, was the major chemerin form in plasma. Like the total chemerin level, the prochemerin level in mouse plasma from either high- or low-fat fed animals was not significantly different at 12 weeks. But at 26 weeks, high-fat fed mice had a dramatically increased level of prochemerin contributing to the increased level of total chemerin in plasma compared to low-fat diet mice. This chemerin increase was accompanied by a significant weight and %fat increase at 26 weeks. These findings are consistent with elevated serum chemerin levels in obese humans and rodents [7,18].

The plasma level of the active chemerin form, mchem156S, was found to be elevated significantly in 12-week, but not in 26-week, high-fat fed mice, suggesting that secretion of physiologically relevant amounts of active chemerin (32% of all chemerin forms in plasma) occurs at a time of active early adipocyte differentiation and adipogenesis [36]. This provides *in vivo* evidence supporting the concept that chemerin/CMKLR1 signaling is critical in the adipocyte differentiation process [8]. It is notable that the other active chemerin form, mchem155F, was only detected at a low level in plasma (≤7.7% of all chemerin forms), and showed no significant difference between different diets and different ages. The modestly active chemerin form, mchem157R, was also elevated in plasma in 12-week high-fat diet mice (~23% of all chemerin forms), suggesting that active mchem156S was most likely generated by sequential cleavages via mchem157R followed by removal of the C-terminal lysine residue. Similar to the human total chemerin ELISA [33], the mouse total chemerin ELISA does not detect the different chemerin forms equipotently, making it very difficult to compare its results with that determined by the specific ELISAs to the different forms.

Similarly to the results reported here in mouse plasma and in earlier studies in human plasma [33,37], chemerin levels in serum, determined by means of commercial ELISAs, were also higher in samples from humans and mice with obesity than in serum from normal humans and mice [17,37–40]. Activity on CMKLR1 transfected cells in both human and mouse serum from obese individuals did not correlate with the observed increased in chemerin levels. Conversion of plasma to serum will change the spectrum of chemerin forms present [22]. As different chemerin forms have different activities, the data from serum samples is consistent with the hypothesis that as in plasma [33] serum from individuals with obesity contains different chemerin forms that are present in different proportions than in serum from individuals with normal BMI.

Since chemerin is an adipokine, and over 90% of adipokines are released by adipose tissue, we investigated the chemerin levels in epididymal and brown fat. Epididymal fat is white adipose tissue that stores energy as triglycerides, while brown adipose tissue is specialized for energy dissipation through nonshivering thermogenesis [36]. Under different metabolic condition such as hyperglycemia and hyperinsulinemea as well as during weight changes and aging, significant changes in the expression of adipokines in the visceral adipose tissue are seen [41–44]. Increased visceral fat accumulation is a risk factor for development of metabolic dysfunction. Chemerin levels in epididymal fat extracts were generally higher than those in brown fat extracts (Fig 6A and 6B), consistent with previous reports that murine chemerin mRNA is highly expressed in liver, white adipose tissue, and placenta [8] and white adipocytes are a source and target for chemerin signaling [18]. Our data suggest that the regulation and expression of chemerin in white adipose tissue is distinct from that in brown adipose tissue, consistent with recent findings [45]. Secretion of TNF-α reduces the secretion of adiponectin from adipocytes [46], consistent with increased inflammation in the white adipose tissue during the onset of obesity reducing the secretion of chemerin in white, but not brown, adipose tissue. The dominant active chemerin form detected at 12 weeks was mchem155F, but not at 26 weeks, in mouse epididymal fat, supporting the hypothesis that active chemerin is involved in early adipocyte differentiation and adipogenesis. In marked contrast to the plasma levels, mchem156S was not detectable in epididymal fat at 12 weeks, suggesting that active chemerin in adipose tissue was not generated by sequential cleavages of prochemerin via mchem157R to mchem156S, but instead by cleavage of prochemerin to mchem155F, likely mediated by chymase [24]. Chymase is expressed in both human and rodent adipose tissues [47,48]. Our data demonstrate that chemerin activation is regulated by different proteolytic cleavage pathways in different tissue compartments.

Active chemerin can drive adipogenesis in cultured 3T3-L1 preadipocytes and bone marrow derived mesenchymal stem cells [8,49] suggesting that it should play a role in obesity. However mice deficient in the gene encoding chemerin [11] or mice deficient in the genes encoding the signaling chemerin receptors, CMKLR1 and GPR1 [18,50], do not have lower body weight or fat % except that due to the change in myogenesis observed in CMKLR1 deficient mice [50]. There was no difference in weight between chemerin deficient and wild type mice when they were fed a high fat diet [11]. In contrast to the minimal effects on weight or fat%, the chemerin, CMKLR1 and GPR1 deficient mice all have differences from wild type mice in glucose metabolism [11,12,18]. In human studies, however, the data is inconsistent about the effects of obesity, diabetes or both on chemerin levels [51].

Adiponectin is a well-characterized adipokine that is down-regulated in the epididymal adipose tissue in the obese state [52,53]. We measured adiponectin levels in mouse plasma as well as in epididymal and brown adipose tissue extracts (Fig 7A–7C) as a marker that changes expression in adipose tissue in response to the mice being fed a high fat diet. The level of adiponectin in blood, as well as in epididymal adipose extracts, decreased in 26-week old mice, especially in obese mice with high fat feeding, confirming that BMI and adiponectin levels are negatively correlated [36]. The adiponectin level in brown adipose tissue extracts, on the other hand, increased in 26-week old mice. In addition, the adiponectin and total chemerin levels in epididymal adipose extracts were positively correlated (Fig 6D). Taken together, chemerin and adiponectin from adipose tissue extracts share a common expression pattern in obesity and might be subject to the same regulatory feedback mechanisms. Plasma levels of chemerin and adiponectin, however, have different expression patterns as an increase in body weight and fat % leads to elevated plasma chemerin but reduced adiponectin.

A shortcoming of our studies is that the activity of the different forms of chemerin was only studied on cells over-expressing CMKLR1. Although human chemerin binding to GPR1 is important for its possible role in regulating adipogenesis [12] and has been shown to signal [54], to date there have been no studies showing the relative potencies of different mouse and human chemerin forms on GPR1. Another issue with our studies is that we did not investigate subcutaneous white adipose tissue that is more susceptible to formation of beige adipocytes [55,56].

**Fig 8:**
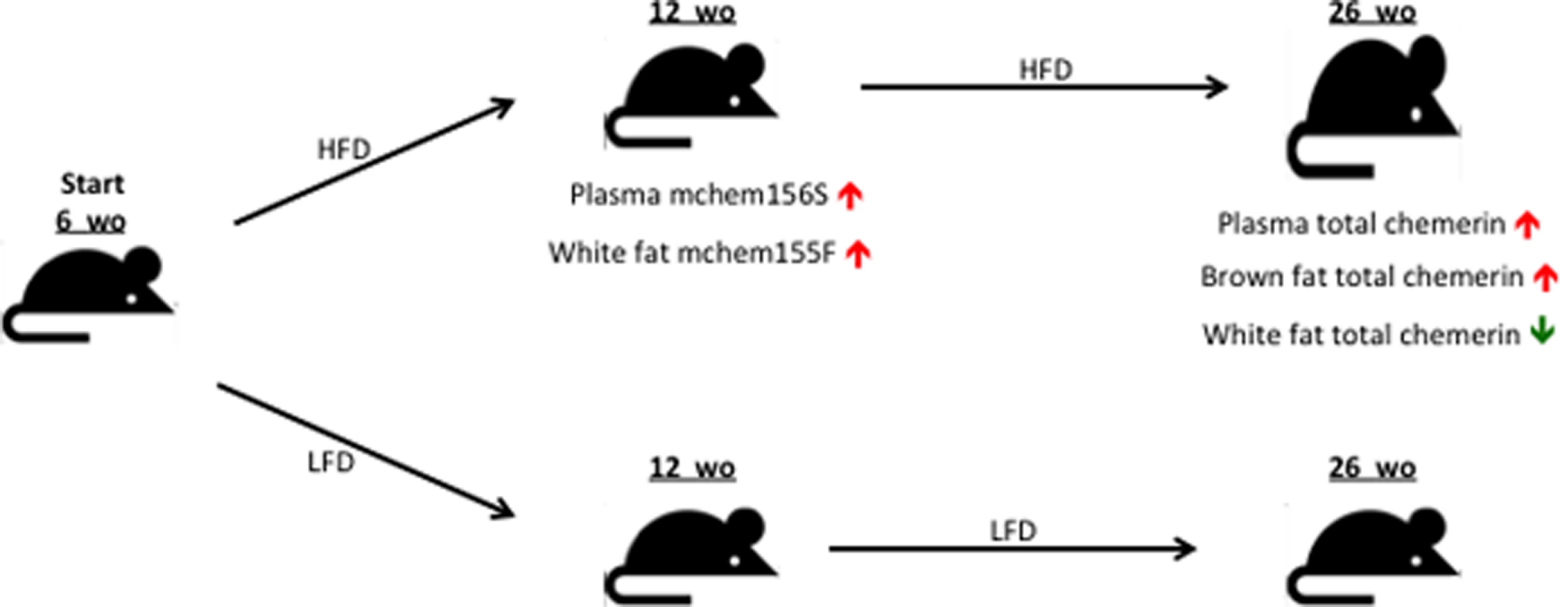
Summary of chemerin changes with diet induced obesity. Mice were started on either a high fat diet (HFD) or low fat diet (LFD) at 6 weeks old (wo) and were sacrificed at 12 wo or 26 wo for analysis of chemerin levels in plasma, epididymal (white) and brown adipose tissue (fat). Mice on HFD were heavier and had higher %fat at 26 wo. Increase in levels shown as red arrows and decrease in levels shown as a green arrow.

In summary, our study provides the first comprehensive characterization of the different mouse chemerin forms and the determination of their levels in a mouse model of obesity *in vivo*, validating the use of the mouse as a model for chemerin studies. We confirmed in our study of mice fed different diets that mouse chemerin plasma levels increased with obesity (Fig 8). In particular, the biologically active mchem156S and mchem155F became elevated during the onset of obesity, the former in plasma and the latter in epididymal adipose tissue. This pattern of increased chemerin and modulation of its biological activity by proteolytic processing is similar to our observations in obese humans. We also demonstrated that chemerin level in epididymal adipose tissue decreased, while its level in brown adipose tissue increased in obese mice, confirming that chemerin gene expression and regulation are distinct in these two adipose tissues. The new set of tools described here now allows studies of chemerin biology in different mouse models.

## Supporting information

S1 Table Primers used for constructing expression vectors. mCHEM-5’Ngo was used as the sense primer, mCHEM-FL-3’Nhe for mchem162K, mCHEM-157R-3’Nhe for mchem157R, hCHEM-3 Nhe-S for mchem156S, hCHEM-3 Nhe-F for mchem155F and mCHEM-154A-3’Nhe for mchem154A were used as anti-sense primers, in which the underlined nucleotides are restriction enzyme sites described in the right column.
(DOCX)

S2 Table Antibodies. Antibodies used in these studies are described.
(DOCX)

S1 Fig Alignment of chemerin protein sequences from different species. Chemerin sequences were extracted from the Ensembl database and compared using Wasabi. Gaps were introduced to improve alignment. Two marsupial species (opossum and Tasmanian devil) have potential initiator Met codons 5 amino acids preceding the consensus initiator Met codon (marked with red arrow). The signal sequence cleavage site is marked with a blue arrow and the basic amino acid at the cleavage site that removes the C-terminal tail to generate active chemerin is marked with a black arrow. Alignment of chemerin protein sequences from different species shows good homology.
(DOCX)

S2 Fig Purification and characterization of mouse chemerin forms. Following centrifugation and filtration, conditioned cell culture medium was applied to an anion exchange column equilibrated with PBS (pH 7.4) and then chemerin proteins were eluted by a gradient of increasing ionic strength. **A**, absorbance at 280 nm (mAU, *blue line*), NaCl gradient from 150 to 500 mM (*green)*, and conductivity (*brown*) of a purification of mchem156S. **B**, Coomassie Blue stained SDS-PAGE analysis of fractions from the anion exchange column of a purification of mchem156S. postculture medium supernatant: S; flow-through: F; wash: W. Fraction numbers are shown. Molecular mass markers are shown on the left. **C,** Coomassie Blue stained SDS-PAGE analysis of purified recombinant mchem161T, mchem157R, mchem156S, mchem155F, and mchem154A. Molecular mass markers are shown on the left.
(PPTX)

S3 Fig MALDI-TOF-MS spectra of mouse chemerin forms. MALDI-TOF-MS spectra of mchem161T (*upper left*), mchem157R (*middle left*), mchem156S (*lower left*), mchem155F (*upper right*) and mchem154A (*middle right*). The molecular mass of the principal peak is indicated.
(PPTX)
