# Supplementary material for: Dynamic and tissue-specific proteolytic processing of chemerin in obese mice
Source: PLoS One. 2018 Aug 30;13(8):e0202780. doi: 10.1371/journal.pone.0202780 (PMC6116994; doi:10.1371/journal.pone.0202780)
Supplement: S1 Table — mCHEM-5’Ngo was used as the sense primer, mCHEM-FL-3’Nhe for mchem162K, mCHEM-157R-3’Nhe for mchem157R, hCHEM-3 Nhe-S for mchem156S, hCHEM-3 Nhe-F for mchem155F and mCHEM-154A-3’Nhe for mchem154A were used as anti-sense primers, in which the underlined nucleotides are restriction enzyme sites described in the right column. (DOCX) [file pone.0202780.s001.docx]

**Supplemental Table 1.** **Primers for constructing expression plasmids encoding different mouse chemerin forms.**

**
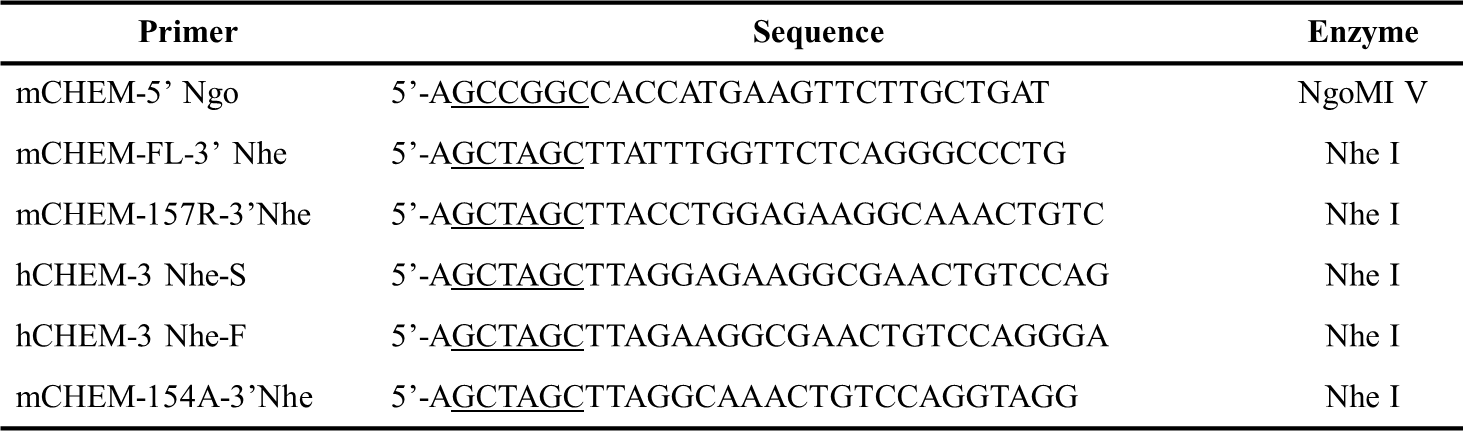
**

mCHEM-5’Ngo was used as the sense primer, mCHEM-FL-3’Nhe for mchem162K, mCHEM-157R-3’Nhe for mchem157R, hCHEM-3 Nhe-S for mchem156S, hCHEM-3 Nhe-F for mchem155F and mCHEM-154A-3’Nhe for mchem154A were used as anti-sense primers, in which the underlined nucleotides are restriction enzyme sites described in the right column.
