# Supplementary material for: Dynamic and tissue-specific proteolytic processing of chemerin in obese mice
Source: PLoS One. 2018 Aug 30;13(8):e0202780. doi: 10.1371/journal.pone.0202780 (PMC6116994; doi:10.1371/journal.pone.0202780)
Supplement: S2 Table — Antibodies used in these studies are described. (DOCX) [file pone.0202780.s002.docx]

**S2 Table: Antibodies**

| **Antibody** | **Source** | **Catalog number** | **RRID** | **Working concentration** |
| --- | --- | --- | --- | --- |
| Anti-mchem162K rabbit polyclonal | raised against peptide ^150^CGQFAFSRALRTK^162^ |  |  | 500 ng/ml |
| Anti-mchem157R rabbit polyclonal | raised against peptide K^151^CGQFAFS^157^ |  |  | 500 ng/ml |
| Anti-hchem157S rabbit polyclonal | Zhao et al, (30) |  |  | 500 ng/ml |
| Anti-hchem156F chicken polyclonal | raised against peptide CZ^151^PGQFAF^156^ |  |  | 500 ng/ml |
| Anti-hchem155A chicken polyclonal | Chang et al, (31) |  |  | 500 ng/ml |
| Anti-mouse chemerin rat monoclonal | R&D Systems | MAB23251 | AB_1964541 | 4 µg/ml |
| HRP-conjugated goat anti-rabbit IgG | Jackson ImmunoResearch Labs | 111-035-003 | AB_2313567 | 100 ng/ml |
| HRP-conjugated goat anti-chicken IgY | Aves Lab | H-1004 | AB_2313517 | 100 ng/ml |
