# Supplementary material for: Dynamic and tissue-specific proteolytic processing of chemerin in obese mice
Source: PLoS One. 2018 Aug 30;13(8):e0202780. doi: 10.1371/journal.pone.0202780 (PMC6116994; doi:10.1371/journal.pone.0202780)
Supplement: S1 Fig — Chemerin sequences were extracted from the Ensembl database and compared using Wasabi. Gaps were introduced to improve alignment. Two marsupial species (opossum and Tasmanian devil) have potential initiator Met codons 5 amino acids preceding the consensus initiator Met codon (marked with red arrow). The signal sequence cleavage site is marked with a blue arrow and the basic amino acid at the cleavage site that removes the C-terminal tail to generate active chemerin is marked with a black arrow. Alignment of chemerin protein sequences from different species shows good homology. (DOCX) [file pone.0202780.s003.docx]

**S1 Figure. Alignment of chemerin protein sequences from different species**

**
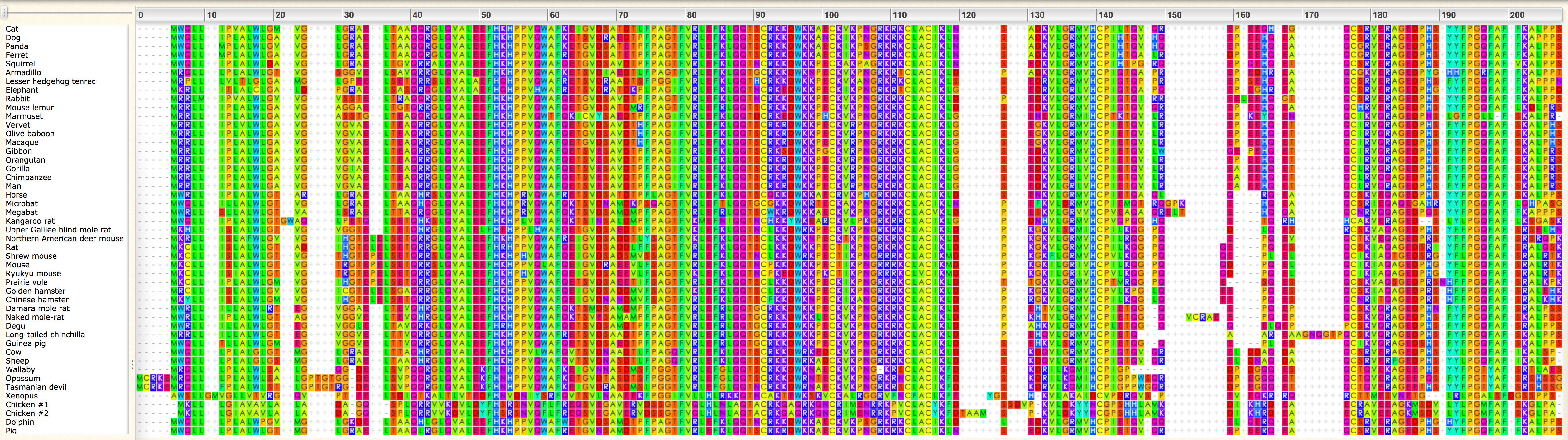
**

Chemerin sequences were extracted from the Ensembl database and compared using Wasabi. Alignment of chemerin protein sequences from different species show good homology
