# Supplementary material for: Dynamic and tissue-specific proteolytic processing of chemerin in obese mice
Source: PLoS One. 2018 Aug 30;13(8):e0202780. doi: 10.1371/journal.pone.0202780 (PMC6116994; doi:10.1371/journal.pone.0202780)
Supplement: S2 Fig — Following centrifugation and filtration, conditioned cell culture medium was applied to an anion exchange column equilibrated with PBS (pH 7.4) and then chemerin proteins were eluted by a gradient of increasing ionic strength. A, absorbance at 280 nm (mAU, blue line), NaCl gradient from 150 to 500 mM (green), and conductivity (brown) of a purification of mchem156S. B, Coomassie Blue stained SDS-PAGE analysis of fractions from the anion exchange column of a purification of mchem156S. postculture medium supernatant: S; flow-through: F; wash: W. Fraction numbers are shown. Molecular mass markers are shown on the left. C, Coomassie Blue stained SDS-PAGE analysis of purified recombinant mchem161T, mchem157R, mchem156S, mchem155F, and mchem154A. Molecular mass markers are shown on the left. (PPTX) [file pone.0202780.s004.pptx]

## Slide 1
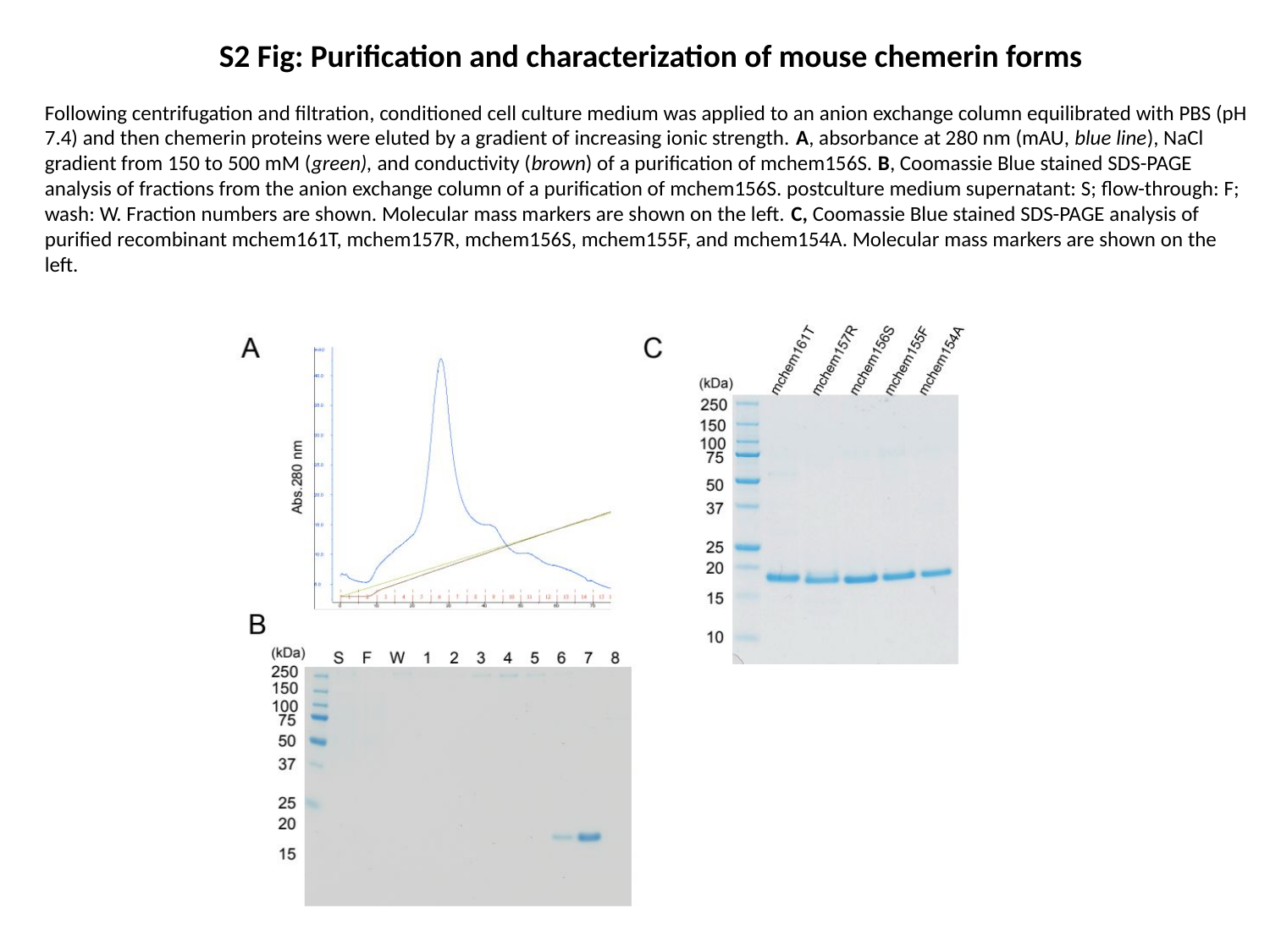

S2 Fig: Purification and characterization of mouse chemerin forms
Following centrifugation and filtration, conditioned cell culture medium was applied to an anion exchange column equilibrated with PBS (pH 7.4) and then chemerin proteins were eluted by a gradient of increasing ionic strength. A, absorbance at 280 nm (mAU, blue line), NaCl gradient from 150 to 500 mM (green), and conductivity (brown) of a purification of mchem156S. B, Coomassie Blue stained SDS-PAGE analysis of fractions from the anion exchange column of a purification of mchem156S. postculture medium supernatant: S; flow-through: F; wash: W. Fraction numbers are shown. Molecular mass markers are shown on the left. C, Coomassie Blue stained SDS-PAGE analysis of purified recombinant mchem161T, mchem157R, mchem156S, mchem155F, and mchem154A. Molecular mass markers are shown on the left.
