# Supplementary material for: Dynamic and tissue-specific proteolytic processing of chemerin in obese mice
Source: PLoS One. 2018 Aug 30;13(8):e0202780. doi: 10.1371/journal.pone.0202780 (PMC6116994; doi:10.1371/journal.pone.0202780)
Supplement: S3 Fig — MALDI-TOF-MS spectra of mchem161T (upper left), mchem157R (middle left), mchem156S (lower left), mchem155F (upper right) and mchem154A (middle right). The molecular mass of the principal peak is indicated. (PPTX) [file pone.0202780.s005.pptx]

## Slide 1
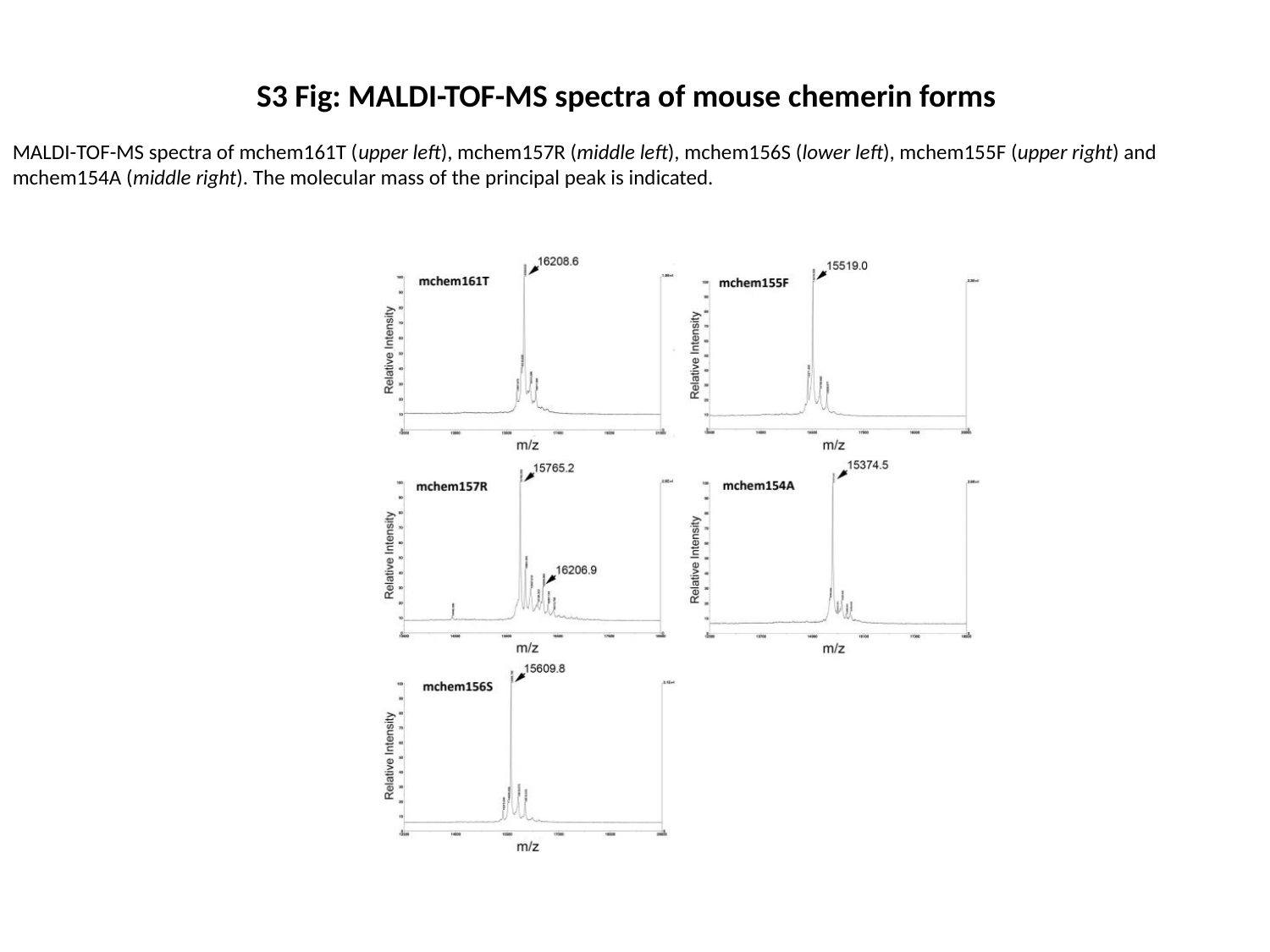

S3 Fig: MALDI-TOF-MS spectra of mouse chemerin forms
MALDI-TOF-MS spectra of mchem161T (upper left), mchem157R (middle left), mchem156S (lower left), mchem155F (upper right) and mchem154A (middle right). The molecular mass of the principal peak is indicated.
